# The complete chloroplast genome of *Cibotium barometz* (Cibotiaceae), an endangered CITES medicinal fern

**DOI:** 10.1080/23802359.2018.1462128

**Published:** 2018-04-12

**Authors:** Shanshan Liu, Zhen Wang, Ting Wang, Yingjuan Su

**Affiliations:** a School of Life Sciences, Sun Yat-sen University, Guangzhou, China;; b College of Life Sciences, Nanjing Agricultural University, Nanjing, China;; c College of Life Sciences, South China Agricultural University, Guangzhou, China;; d Research Institute of Sun Yat-sen University in Shenzhen, Shenzhen, China

**Keywords:** *Cibotium barometz*, chloroplast genome, endangered CITES medicinal fern, phylogenetic analysis

## Abstract

The complete chloroplast (cp) genome sequence of *Cibotium barometz*, an endangered CITES medicinal fern, was determined by Illumina sequencing. Its genome is a circular molecule of 166,027 bp in length, including a pair of inverted repeats (IRs) of 29,158 bp, a large single-copy (LSC) region of 85,665 bp, and a small single-copy (SSC) region of 22,046 bp. The genome encodes 118 unique genes, involving 84 protein-coding genes, 28 tRNA genes, four rRNA genes, and two pseudogenes. Maximum likelihood tree showed that *C. barometz* was closely related to *Alsophila spinulosa*. This investigation lays solid foundations for endangered medicinal resource conservation and fern phylogenetic studies.

*Cibotium barometz* (L.) J. Sm is a large terrestrial tree fern with fronds up to 2 metres tall in Cibotiaceae (Smith et al. 2006), previously in Dicksoniaceae. As an indicator species of acidic soils, it is widely distributed in south subtropical and tropical regions, such as Guangdong, Guangxi, Guizhou, Sichuan, and Yunnan in China (Zhang and Nishida 2013). This fern prefers warm humid environments at elevations up to 1600 m (Zhang and Nishida 2013). Its most prominent feature reflects in stump-like rhizome covered by long, soft, golden-yellow hairs, showing like a golden-haired dog. The species is not only an ornamental fern, but also a famous traditional Chinese herbal plant known as ‘Gouji’ with anti-inflammation and anti-osteoporosis activities (Cuong et al. 2009; Wu and Yang 2009; Zhao et al. 2011). Due to uncontrolled collection, *C. barometz* is listed as an endangered plant of National Protection Grade II, and in Appendix II of the Convention on International Trade in Endangered Species of Wild Fauna and Flora as well (CITES 2017). In addition, *Cibotium* is subjected to much confusion and revision (Zhang and Nishida 2013). Hence, the acquirement of whole chloroplast (cp) genome of *C. barometz* will contribute to resource protection and phylogenetics.

The plant sample was harvested from South China Botanical Garden, Chinese Academy of Sciences. Voucher specimen is held by the Herbarium of Sun Yat-sen University (SYS; voucher: *SS Liu 20161011*). Genomic DNA was extracted from fresh leaves using Tiangen Plant Genomic DNA Kit (Tiangen Biotech Co., Beijing, China). Approximately 300–500 bp paired-end library was constructed and sequenced in Hiseq 2500 platform (Illumina Inc., San Diego, CA, USA). After adapters or low-quality reads were removed by Trimmomatic (Bolger et al., 2014), a genome was obtained through *de novo* assembly using Velvet v1.2.07 (Zerbino and Birney 2008). DOGMA (Wyman et al. 2004) and tRNAscan-SE 1.21 (Schattner et al. 2005) were applied to perform annotation. Maximum-likelihood analysis including 11 ferns was conducted based on complete cp genome sequences using RAxMLv.8.0 (Stamatakis 2014) with 1000 bootstrap replicates. *Lygodium japonicum* was selected as outgroup.

The complete chloroplast genome of *C. barometz* is 166,027 bp in length, with a pair of IR regions of 29,158 bp that separate an LSC region of 85,665 bp and an SSC region of 22,046 bp (GenBank accession number: MH105066). It encodes 131 genes, of which 118 genes are unique, including 84 protein-coding genes (PCGs), 28 tRNA genes, four rRNA genes, and two pseudogenes. Thirteen genes are duplicated, containing four rRNA genes (*rrn5*, *rrn4.5*, *rrn23*, and *rrn16*), five tRNA genes (*trnN*-*GUU*, *trnH*-*GUG*, *trnI*-*GAU*, *trnA*-*UGC*, and *trnR*-*ACG*), three PCGs (*psbA*, *rps7*, and *rps12*), and one pseudogenes (*ycf2*). Nine PCGs and five tRNA genes have one intron, whereas two PCGs and one trans-splicing gene contain two introns. The overall GC content is 41.7%. ML tree revealed that *C. barometz* was sister to *Alsophila spinulosa* (Figure 1). The complete chloroplast genome of *C. barometz* lays solid foundations for endangered medicinal resource conservation and fern phylogenetic studies.

**Figure 1.**
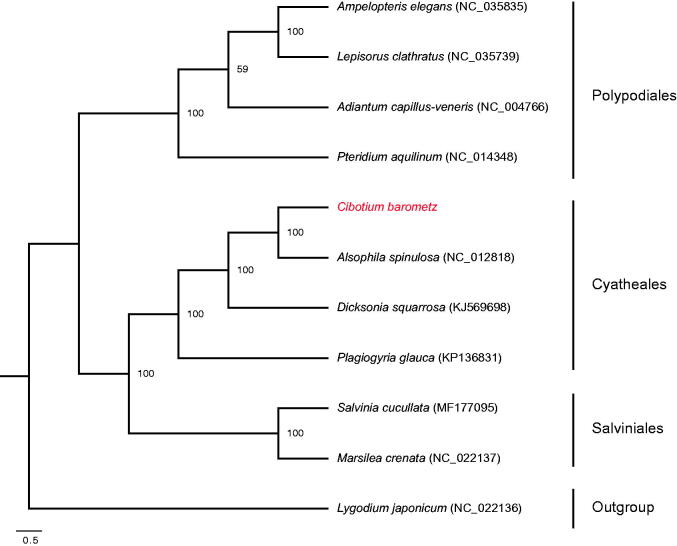
Maximum likelihood phylogenetic tree inferred from whole chloroplast genome sequences of 11 ferns with *Lygodium japonicum* as outgroup. Bootstrap support values based on 1000 replicates are indicated in each node.
